# Effects of WC-17Co Coating Combined with Shot Peening Treatment on Fatigue Behaviors of TC21 Titanium Alloy

**DOI:** 10.3390/ma9110865

**Published:** 2016-10-25

**Authors:** Dongxing Du, Daoxin Liu, Xiaohua Zhang, Jingang Tang, Baoli Meng

**Affiliations:** 1 Institute of Corrosion and Protection, School of Aeronautics, Northwestern Polytechnical University, Xi’an 710072, China; yhzhangxh@nwpu.edu.cn; 2 Institute of Machinery Manufacturing Technology, China Academy of Engineering Physics, Mianyang 621999, China; jingangtang@163.com; 3 Xi’an Aircraft International Corporation, Xi’an 710089, China; liuyunzhy@163.com

**Keywords:** WC-Co coating, TC21 titanium alloy, fatigue, shot peening, residual stress, toughness

## Abstract

The improvement and mechanism of the fatigue resistance of TC21 high-strength titanium alloy with a high velocity oxygen fuel (HVOF) sprayed WC-17Co coating was investigated. X-ray diffraction (XRD) and the corresponding stress measurement instrument, a surface roughness tester, a micro-hardness tester, and a scanning electron microscope (SEM) were used to determine the properties of the HVOF WC-17Co coating with or without shot peening. The fatigue behavior of the TC21 titanium alloy with or without the WC-17Co coating was determined by using a rotating bending fatigue testing machine. The results revealed that the polished HVOF sprayed WC-17Co coating had almost the same fatigue resistance as the TC21 titanium alloy substrate. This resulted from the polishing-induced residual surface compressive stress and a decrease in the stress concentration on the surface of the coating. Moderate-intensity shot peening of the polished WC-17Co coatings resulted in significant improvement of the fatigue resistance of the alloy. Furthermore, the fatigue life was substantially higher than that of the substrate, owing to the deep distribution of residual stress and high compressive stress induced by shot peening. The improved surface toughness of the coating can effectively delay the initiation of fatigue crack propagation.

## 1. Introduction

Ti-6Al-2Sn-2Zr-3Mo-1Cr-2Nb, referred to as TC21 in China, has emerged as a novel high-strength titanium alloy. This alloy is used in the aviation industry owing to its remarkable characteristics: high strength-to-weight ratio, outstanding toughness, and low crack propagation rate. In fact, the overall performance and engineering application value of TC21 renders this alloy especially suitable for machining aircraft landing gear [1,2]. However, the poor wear resistance of titanium alloys limit their use under friction conditions. The wear resistance of metallic materials can be significantly increased by using high velocity oxygen fuel (HVOF) spraying WC-Co coating technology. This method prevents mesh micro-cracking of the hard chrome plating layer, and is environmentally friendly. Therefore, HVOF technology has significant potential for improving the wear resistance of aircraft landing gear parts [3,4,5,6,7]. Fatigue failure, the main damage mode of aircraft landing gear parts, results from the anti-fatigue properties of WC-Co hard coatings deposited on metal substrates. Owing to its high surface-notch sensitivity, the adverse effect of the hard coating is especially pronounced in the case of this high-strength titanium alloy. The anti-fatigue performance of HVOF WC-Co coatings can be improved via polishing, but retention of the base-metal fatigue resistance is often difficult [3,4,5,8,9,10].

The anti-fatigue performance of metallic materials can be effectively improved via shot peening. In fact, the fatigue and fretting fatigue resistance of titanium alloys can be significantly improved by combining shot peening with plasma nitriding, carbonizing, and surface alloying [8,11,12,13]. However, the fatigue resistance of the HVOF WC-17Co coating obtained through a shot peening pretreatment was lower than that of the TC21 high-strength titanium alloy [1]. Applying a suitable shot peening after-treatment to the polished HVOF WC-Co coatings constitutes a promising method for improving the anti-fatigue performance, but remains, however, unexplored.

Therefore, the effect of HVOF WC-17Co coatings and shot peening on the fatigue behavior of the TC21 high-strength titanium alloy was investigated in this work. The goal of this study is to provide ideas that lead to improved fatigue performance of the HVOF WC-Co hard coating and, in turn, of the high-strength titanium alloy parts.

## 2. Materials and Methods

### 2.1. Materials and Spraying Powder

The chemical composition of the TC21 high-strength titanium alloy is given as follows: 6.29% Al, 2.32% Sn, 2.24% Zr, 2.59% Mo, 1.74% Cr, 2.01% Nb, 0.026% C, 0.002% H, 0.077% O, 0.008% N, with the balance being Ti. Prior to the experiments, the specimens were annealed at 910 °C for 1 h and 550 °C for 6 h, then air-cooled to room temperature. The mechanical properties were measured at room temperature, and values of 1171 MPa, 1130 MPa, 11%, and 56% were obtained for the ultimate tensile strength, yield strength, elongation, and area reduction, respectively.

**Figure 1:**
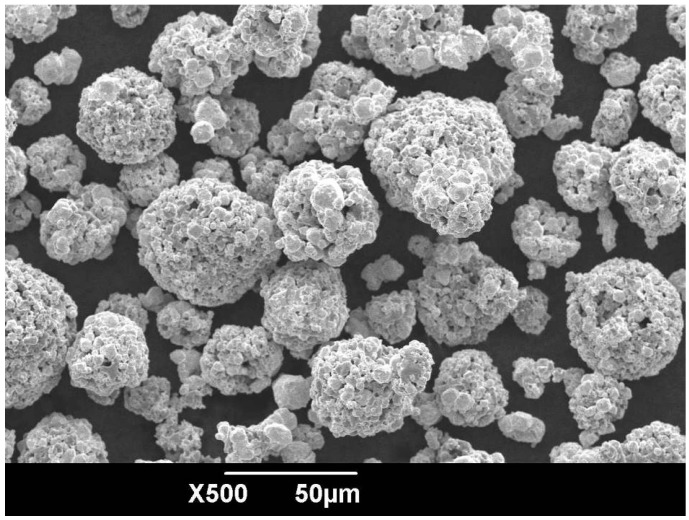
SEM morphology of WC-17Co powder.

The WC-17Co sprayed powder (Diamalloy 2005NS; Sulzer Metco Corp., Allschwil, Switzerland) consists of 5.5–45 μm sized particles, whose morphology is shown in Figure 1.

### 2.2. Research Methods

The TC21 alloy was subjected to a shot peening pretreatment that was performed with the aim of improving the anti-fatigue performance of the titanium alloy substrate, and the bonding strength of the HVOF WC-17Co coatings. The process parameters used for the pretreatment were based on our previous studies [1]. Furthermore, the Z300 ceramic shots used during the treatment have an average diameter of 0.30 mm and consist of 68% ZrO_2_ and 32% SiO_2_. A peening intensity of 0.20 mmA (Almen intensity) was used during the treatment. The ratio of coverage and the shot blasting angle were 100% and 90°, respectively. Hereafter, the shot peening pretreated samples of the TC21 alloy are referred to as SP. The HVOF WC-17Co coating was deposited by using a Sulzer Metco DJ-2700 spray gun (Sulzer Metco Corp., Allschwil, Switzerland), using the process parameters shown in Table 1. The pretreated samples with HVOF WC-17Co coatings are referred to as SP + H. Moreover, HVOF WC-17Co coatings that were polished by grinding with various grades of diamond abrasive belts, are referred to as SP + H + DP. Polished coatings that were subsequently shot peened are referred to as SP + H + DP + SP. Coatings shot-peened at Almen intensities of 0.10 mmA, 0.20 mmA, and 0.30 mmA are referred to as SP + H + DP + SP1, SP + H + DP + SP2, and SP + H + DP + SP3, respectively. Basic performance tests were performed on 30 mm × 30 mm × 10 mm coated specimens. The WC-17Co coatings were 55 μm thick.

The surface roughness (Ra) was measured by using a Surf Test SJ-301 (Mitutoyo Corp., Kawasaki, Japan). Furthermore, a micro-hardness tester was used to measure the Knoop hardness during 20 s loading of the films at loads ranging from 0–0.98 N. The residual stress was measured by using a D/MAX 2200 PC-type computer-assisted X-ray stress analyzer (Rigaku Corporation, Tokyo, Japan). The measurements were performed by using the roll method that incorporates a fixed-Ψ0 scan method (Ψ0 values of 0°, 15°, 30°, and 45° were used); the full-width at half-maximum was used to determine the position of the peak. These measurements were performed on a 2 mm × 2 mm irradiated region, using Cu (Kα) target radiation, a tube voltage and current of 40 kV and 30 mA, respectively, and parallel beam configuration. The stress constants of Ti and the WC coating were −277 MPa/° at the (213) diffraction crystal plane and –1050 MPa/° at the (301) diffraction crystal plane. The residual stress distribution in the coating was determined via the delamination method. A JSM-6390A scanning electron microscope (SEM) (JEOL, Ltd., Tokyo, Japan) was used to examine the coating structure and morphology. In addition, the coating composition and toughness were determined via energy dispersive spectroscopy (EDS) and by using a cyclic in-house press–press device [14], respectively.

**Figure 2:**
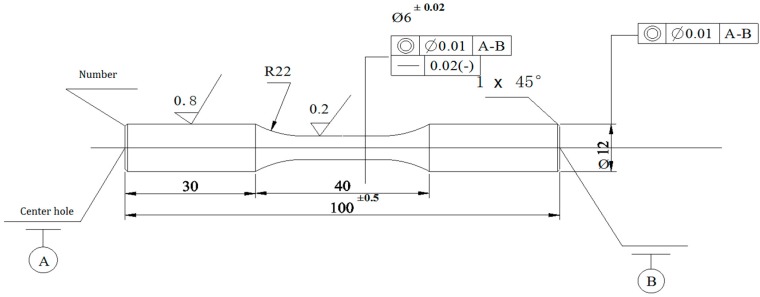
Schematic diagram of fatigue specimen size (unit mm).

A PQ-6-type rotary bending fatigue testing machine (Lanzhou Institute of Chemical Physics, Lanzhou, China) was used for the fatigue test. The shape and dimensions of the fatigue specimens are shown in Figure 2. Testing was conducted at room temperature and a frequency and stress ratio (R) of 50 Hz and −1, respectively. The specimen surface of the alloy was prepared via longitudinal polishing (Ra = 0.08 μm) and the fatigue tests were conducted after the spraying treatment. Based on our previous work [1], a maximum cyclic stress of 700 MPa was used during testing. The sprayed HVOF WC-17Co coating was ~55 μm thick. Five specimens were tested for each condition, and the fatigue resistance of the samples was determined by assessing the average fracture life. In addition, the surface morphology and fracture characteristics were determined via SEM.

## 3. Results

### 3.1. Characteristics

**Figure 3:**
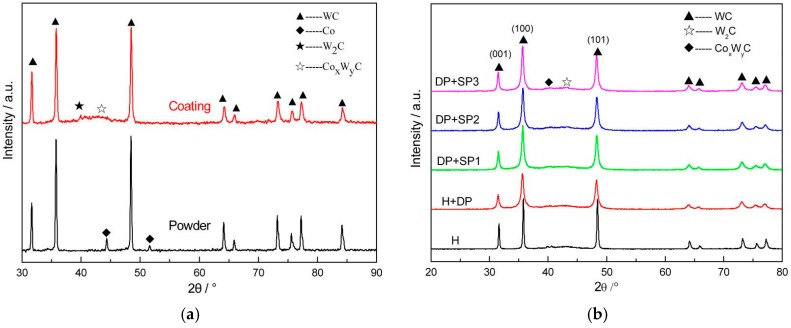
XRD pattern of coatings with different treatments: (**a**) WC-17Co powder and sprayed coating; (**b**) Coatings with polishing and SP treating.

Figure 3 shows the X-ray diffraction (XRD) spectra of WC-17Co powder, the polished and shot peened HVOF WC-17Co coatings. In the Figure 3a, a phase of Co is clearly shown in the powder, while it disappears in the coating spectrum that consists of W_2_C and metastable Co_x_W_y_C phases. Furthermore, the diffraction peak of the coating is wider than that of the powder, which indicates that the coating has microcrystalline characteristics. In addition, a decarbonization phenomenon of WC can be hardly observed in this process of HVOF WC-17Co, which indicates that the increased HVOF jet velocity has inhibited the decomposition and oxidation of WC.

In the Figure 3b, WC constitutes the main phase of the coatings. In other words, the phase composition of the coatings was maintained, even after polishing and shot peening. However, the diffraction peak of the WC-17Co coatings is broader than that of the polished and shot-peened coatings. The higher the shot peening intensity, the significantly wider the XRD diffraction peak. This indicates that the compressive residual stress is introduced in the coatings treated by polishing and shot peening. The grain structure of coatings can also be refined by shot peening.

**Figure 4:**
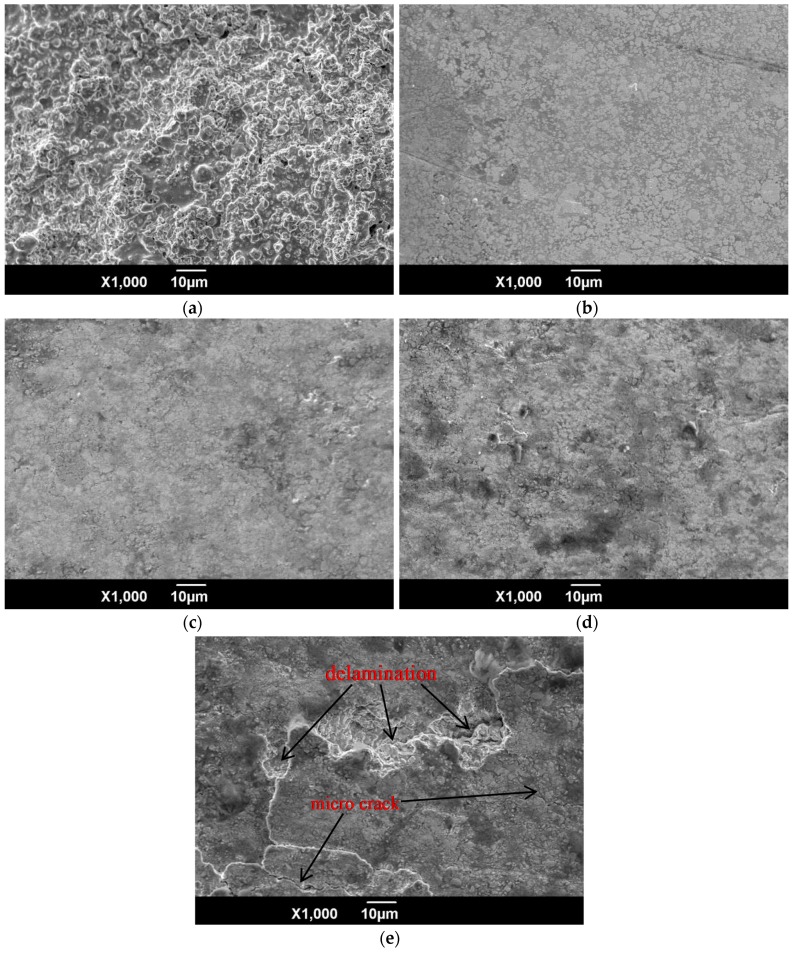
SEM micrographs of specimens with different surface treatments: (**a**) SP + H; (**b**) SP + H + DP; (**c**) SP + H + DP + SP1; (**d**) SP + H + DP + SP2; and (**e**) SP + H + DP + SP3.

The SEM images in Figure 4 show the morphologies of the coatings subjected to various surface treatments. The surface of each coating is rough and porous, as shown in Figure 4a. However, the surface of the polished coating is very smooth, and there were no obvious holes (Figure 4b). The surface morphology of the moderately shot-peened specimens (SP1 and SP2) is very similar to that of the polished specimen (see Figure 4c,d). Excessive shot peening, as in the case of specimen SP3, results in the formation of micro-cracks in, and delamination of, the coatings (see Figure 4e). This is attributed to the difference in hardness between the ceramic ball (700 HK0.98) and the coatings (1800 HK0.98). Therefore, the slight difference in the surface morphology of the polished and moderately shot-peened specimens (SP1 and SP2) is attributed to the low hardness of the low-impact-energy ceramic shot. The impact energy of the ball increases, however, with increasing shot peening intensity. For example, in the case of specimen SP3, the shot peening treatment is similar to the impact fatigue process, in which a ball impacts on the coatings. High intensity results in cracking and delamination on the surface of the coatings, and therefore has a negative effect on the fatigue performance of the titanium alloy.

**Figure 5:**
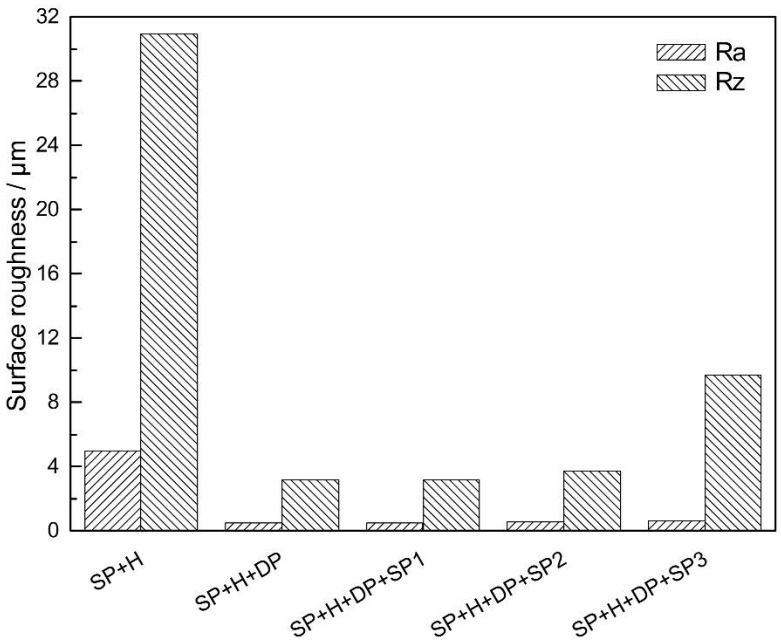
Surface roughness values of specimens with different surface treatments.

The surface roughness values of the coatings, subjected to different surface treatments, are compared in Figure 5. As the figure shows, the Ra and Rz values of the polished coatings are significantly lower than those of the HVOF WC-17Co coatings, whose roughness values are quite high. The parameter Ra describes the average deviation in surface height from the surface roughness profile mean line, while the parameter Rz quantifies the average height from the five highest peaks and five lowest valleys of a surface [15].

The surface of the moderate intensity shot-peened sample (SP2) is only slightly rougher than that of its low-intensity counterpart (SP1). However, surface cracking and delamination occurred in the case of the high-intensity shot-peened coating (SP3). The surface roughness (especially the Rz value) of SP3 is therefore significantly higher than those of SP1 and SP2, as evidenced by the surface morphology (Figure 4e).

**Figure 6:**
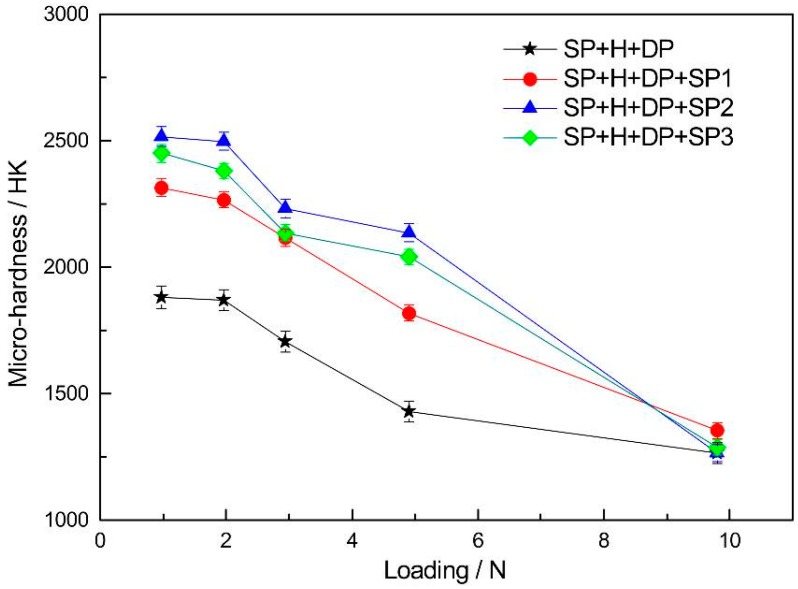
Surface hardness of coatings with different treatments along with loading changing.

Figure 6 shows the micro-hardness as a function of the indentation load applied to the HVOF WC-17Co coatings subjected to different surface treatments. For the same load (especially low loads), the hardness of the polished coatings (SP + H + DP) is lower than that of the polished specimens that underwent a shot peening after-treatment (SP + H + DP + SP). All three types of shot peening treatment resulted in improved surface hardness of the coatings. The hardness increased initially and decreased thereafter, with increasing intensity. Furthermore, hardness of specimen SP2 is higher than that of SP1 (~2516 HK0.98 vs. ~2315 HK0.98). The increase in the hardness of the coatings with shot peening is attributed mainly to the surface residual compressive stress (induced by shot peening) and work hardening of the ductile Co phase of the coatings. However, the hardness of the coated samples all decreased with increasing load. This results from the low hardness of the titanium alloy substrate, i.e., the indentation depth increases with increasing load. The hardness of the SP3 samples (SP + H + DP + SP3) is lower than that of the SP2 samples (SP + H + DP + SP2). This results from surface cracking and delamination damage, induced by SP3 shot peening, and the consequent residual stress relaxation. The high surface hardness of the WC-17Co coatings is conducive for a high resistance to fatigue crack initiation.

**Figure 7:**
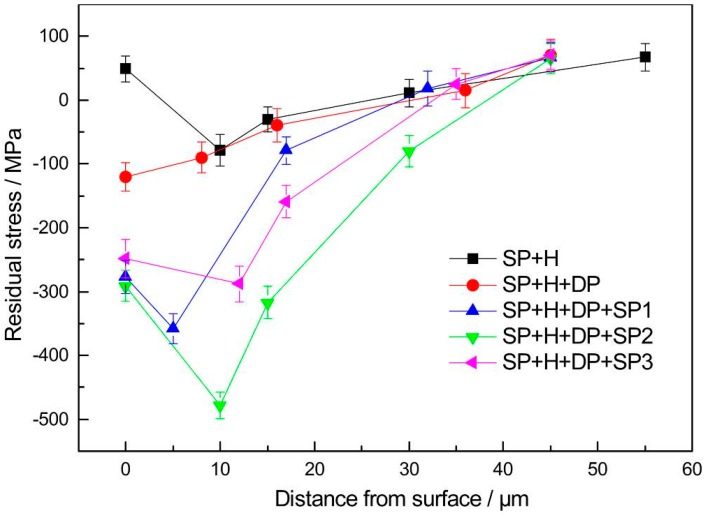
Results of residual stress distribution along cross section of coating with different treatments.

Figure 7 shows the residual stress distributions along the depth in the surfaces of the HVOF WC-17Co coatings. A tensile stress of 49 MPa is present in the coatings that did not undergo a polishing treatment. This residual tensile stress can promote fatigue crack initiation. However, there is significant relaxation of the residual compressive stress in the sub-surface. This stress was induced by the shot peening pretreatment, owing to the high temperature reached during the HVOF process. A compressive stress of −120 MPa can be induced at short distances from the surface of the polished WC-17Co coatings. In fact, the magnitude of the compressive stress and depth of the residual distribution in the polished WC-17Co coatings can be increased by varying the intensity of shot peening. Improved fatigue performance of the titanium alloy substrate can therefore be realized. However, the stress distribution in the coatings varies with the intensity of shot peening. For example, the maximum residual stress value (−478 MPa, 10 μm from the surface), and the largest depth of residual stress distribution are obtained in the case of the moderate intensity shot-peened (SP + H + DP + SP2) sample. The low- and high-intensity shot-peened (SP + H + DP + SP1 and SP + H + DP + SP3) samples have maximum residual stress values of −358 MPa and −288 MPa, which are attributed to insufficient and excessive shot peening intensity, respectively. Therefore, during SP3, cracking and delamination occur readily on the surface of the coatings, thereby leading to relaxation of the residual compressive stress.

### 3.2. Toughness

**Figure 8:**
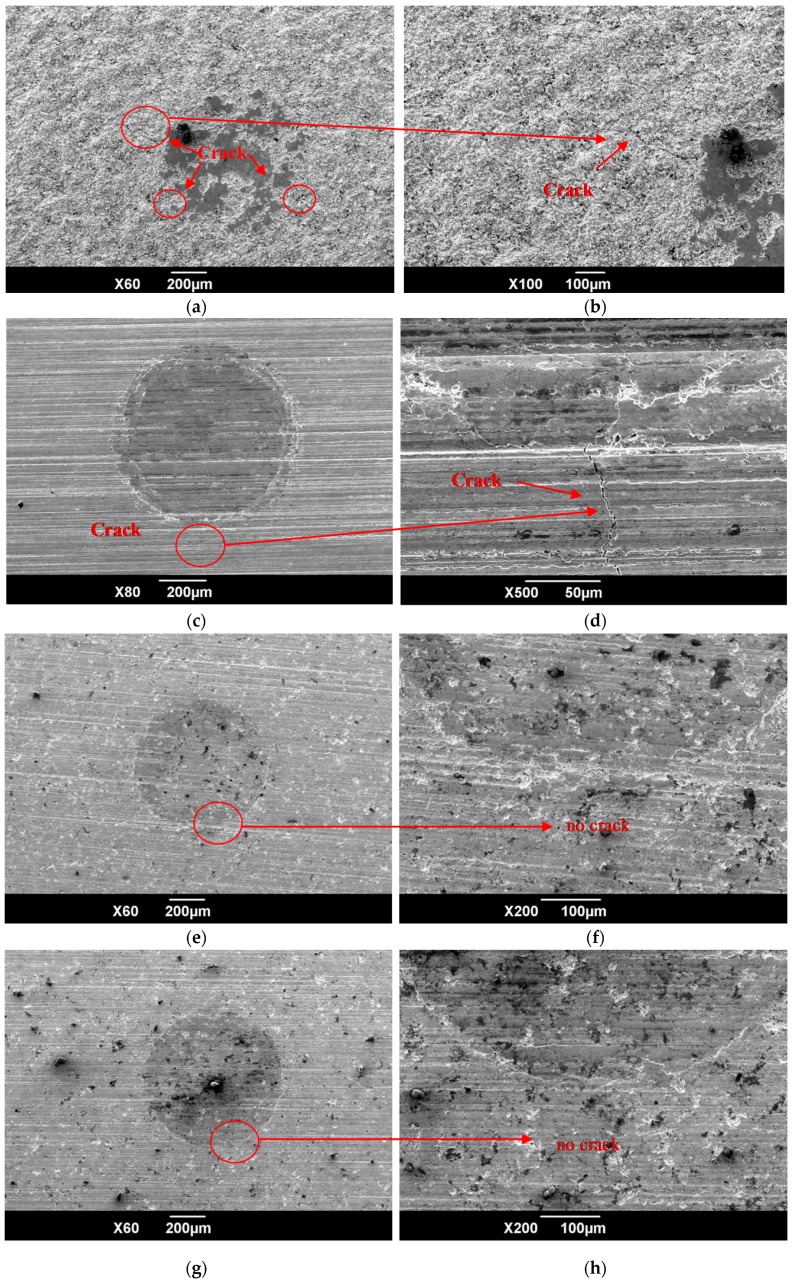

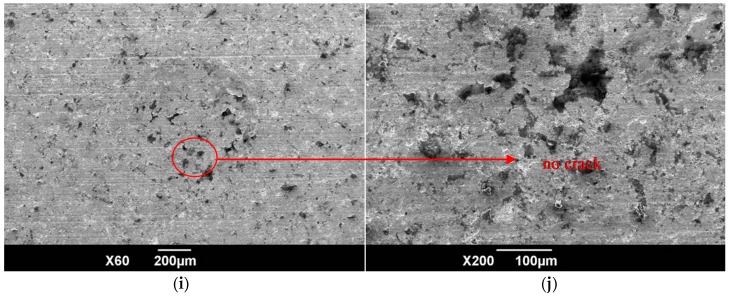
Experiment results of repeated impact for coatings: (**a**) SP + H (existing cracks); (**b**) SP + H enlarged image (existing cracks); (**c**) SP + H + DP (existing cracks); (**d**) SP + H + DP enlarged image (existing cracks); (**e**) SP + H + DP + SP1 (no crack); (**f**) SP + H + DP + SP1 enlarged image (no crack); (**g**) SP + H + DP + SP2 (no crack); (**h**) SP + H + DP + SP2 enlarged image (no crack); (**i**) SP + H + DP + SP3 (no crack); and (**j**) SP + H + DP + SP3 enlarged image (no crack).

The HVOF WC-17Co coatings were subjected to repeated impact tests (10,000 cycles at a load of 400 N), using a hard alloy ball; the resulting surface morphology of the coatings is shown in Figure 8. The untreated HVOF WC-17Co coatings were extremely brittle and rough and hence significant micro-cracking occurred (Figure 8a,b) in these coatings. The degree of stress concentration and the crack density of the polished coatings were lower than those of their unpolished counterparts. Large cracks (Figure 8c,d) formed, however, since the toughness of the coatings changed only slightly with polishing. In contrast, the toughness of the polished coatings increased significantly with shot peening after-treatments and hence, the surface of these coatings remained crack-free even after repeated impact. This crack resistance is attributed to the large and deep residual distribution, induced by shot peening after-treatments, of the polished coatings.

### 3.3. Fatigue Life

**Figure 9:**
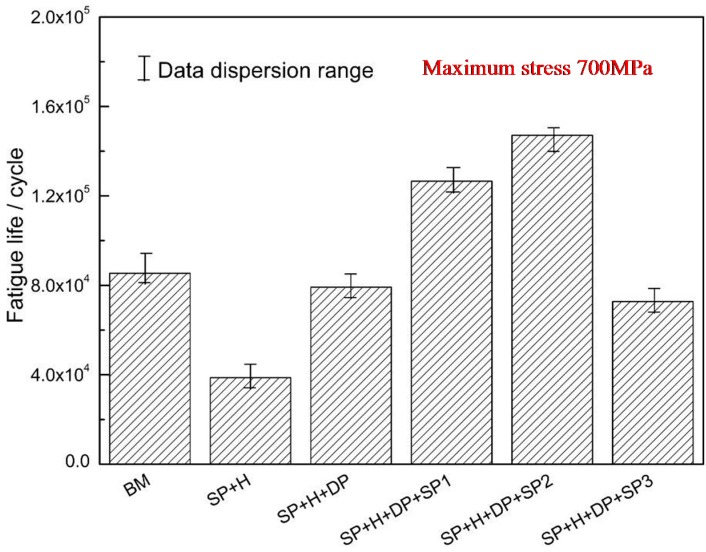
Fatigue life comparison of TC21 specimens with different surface state.

The fatigue life of titanium alloy TC21 with coatings, subjected to various treatments, is shown in Figure 9; the base material (BM) denotes the TC21 titanium alloy substrate. As the figure shows, the fatigue life of coatings that underwent a shot peening pre-treatment (SP + H) is 55% lower than that of the BM samples. This may be attributed to the low toughness, high surface roughness, porosity and residual tensile stress, which are detrimental to the fatigue resistance of the substrate. The fatigue life of the polished coatings (SP + H + DP) is similar to that of the BM samples. This indicates that the notch effect, resulting from the rough surface of HVOF WC-17Co coatings, plays an important role in the fatigue resistance of the TC21 titanium alloy. The fatigue life of the substrate can be improved by shot peening. However, the fatigue life of the polished WC-17Co coatings, shot peened at various intensities, exhibits different trends. The fatigue life of the moderate intensity shot-peened (SP2) samples is 72% higher than that of the BM samples, whereas the fatigue life of the low-intensity counterparts (SP1) is only 48% higher. In contrast, the fatigue life of the high intensity shot-peened (SP3) samples is 48% and 15% lower than that of the polished coatings and BM samples, respectively. This indicates that moderate-intensity shot peening can significantly improve the anti-fatigue performance of WC-17Co coatings. The goal of improving both the wear resistance and anti-fatigue performance of the TC21 high-strength titanium alloy is realized. However, hard WC-17Co coatings crack and delaminate at excessively high intensities of shot peening; high-intensity shot peening is therefore detrimental to the fatigue resistance of these coatings.

### 3.4. Fatigue Fracture Morphology

**Figure 10:**
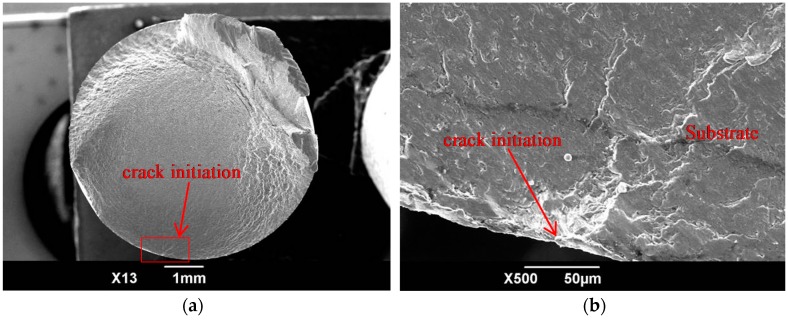

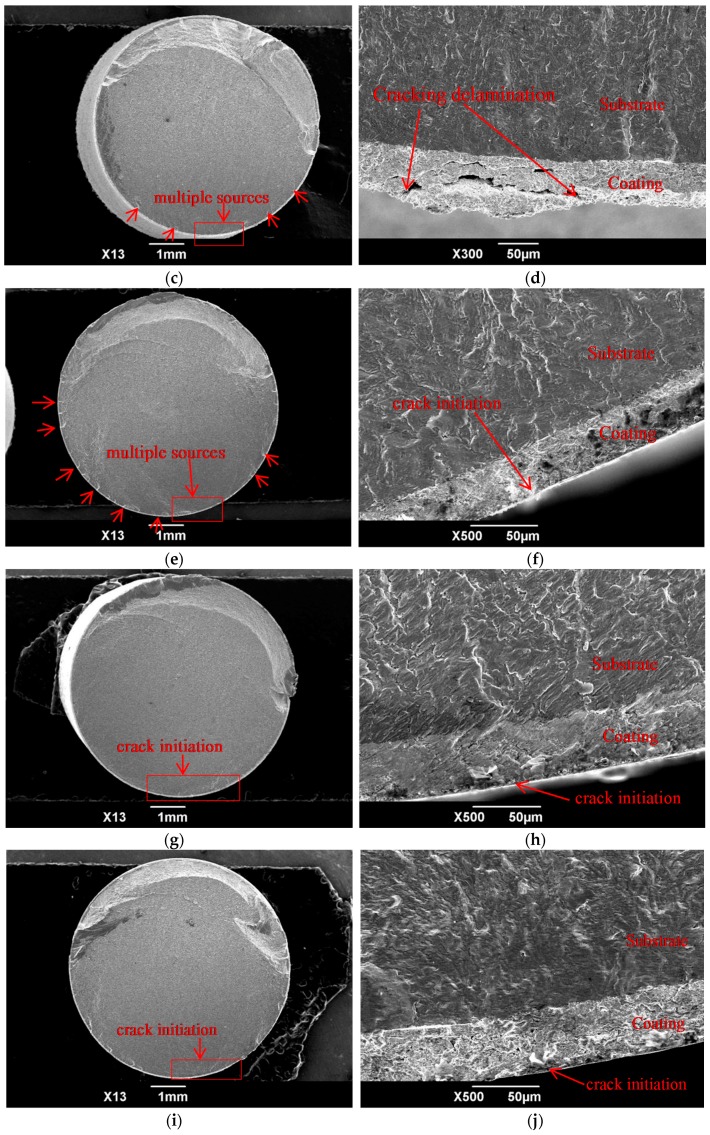

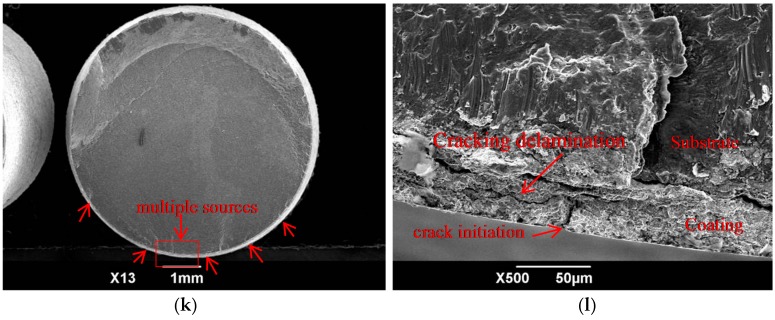
Morphologies of fracture and crack initiation sites of TC21 samples with different surface states: (**a**) BM; (**b**) BM crack initiation site; (**c**) SP + H; (**d**) SP + H crack initiation site; (**e**) SP + H + DP; (**f**) SP + H + DP crack initiation site; (**g**) SP + H + DP + SP1; (**h**) SP + H + DP + SP1 crack initiation site; (**i**) SP + H + DP + SP2; (**j**) SP + H + DP + SP2 crack initiation site; (**k**) SP + H + DP + SP3; and (**l**) SP + H + DP + SP3 crack initiation site.

The fatigue-fracture morphology of the TC21 titanium alloy, subjected to various treatments, is shown in Figure 10. As the figure shows, at maximum amplitude of alternating load, a single fatigue crack is initiated from the surface of the TC21 titanium alloy substrate (i.e., BM). The crack originates from a relatively smooth region of the BM samples and crack propagation is sufficient (Figure 10a,b). The fatigue crack at the immediate surface of the WC-17Co coatings subjected to a shot peening pre-treatment (SP + H), is initiated at multiple sources (Figure 10c,d); these sources result from the ease of crack initiation on the rough surface. In addition, the coatings undergo cohesive failure. The fatigue cracks in the polished (SP + H + DP) coatings also originated from multiple sources (Figure 10e,f). These cracks were initiated at the top surface of the coatings, and then propagated to the interface between the coatings and the substrate, thereby leading to overload fracture of the alloy. The cracks in the polished low intensity (SP1) and moderate intensity (SP2) shot-peened coatings were also initiated at the top surface (Figure 10g–j), albeit from a single source; these cracks propagate extensively. Moreover, these coatings do not undergo cohesive failure and, hence, have a longer fatigue life than the SP+H samples. High-intensity (SP3) shot peening of the polished WC-17Co coatings resulted, however, in cracking or delamination (Figure 4e), decohesion of the coatings, and subsequent separation of the coating/substrate interface (Figure 10k,l). Fatigue cracks were then easily initiated, thereby leading to a decrease in the fatigue resistance.

## 4. Discussion

In summary, the fatigue resistance of the polished WC-17Co coating can be significantly improved. The toughness of these coatings can be improved via the shot peening process, and the corresponding fatigue resistance is higher than those of the polished coating and the TC21 titanium alloy substrate samples (Figure 9). The improved fatigue property of the polished WC-17Co coatings, subjected to a shot peening after-treatment, results from surface hardening and a compressive residual stress that inhibit fatigue crack initiation and retard fatigue crack propagation [16,17,18], respectively.

The results show that the shot peening intensity has significant influence on the fatigue resistance of the WC-17Co coating on titanium alloy. A low-magnitude, shallow distribution of the residual compressive stress field was induced by low-intensity shot peening (SP1). Therefore, the most effective anti-fatigue performance of the coatings was not realized with this treatment. The fatigue performance of the high-intensity shot-peened (SP3) coatings was inferior to that of the titanium alloy substrate. Cracking and delamination of the coatings, owing to the high impact energy of the treatment, resulted in relaxation of the residual compressive stress. Fatigue cracks were easily formed in these coatings, and crack propagation was extensive [16,17,18]. A smooth surface (Figure 4 and Figure 5) and significant hardening (Figure 6) were realized via moderate shot peening (SP2). In fact, the highest magnitude and distribution of the compressive residual stress field were obtained and, hence, crack initiation and propagation were delayed. The fatigue resistance of these specimens was therefore significantly higher than that of the TC21 titanium alloy substrate.

## 5. Conclusions

(1) The fatigue resistance of the TC21 titanium alloy with HVOF WC-17Co coatings that underwent a shot peening pre-treatment, was lower than that of the titanium alloy (i.e., the base material). This resulted from the high hardness, low toughness, rough surface, and the surface residual stress of the coatings. The samples with polished coatings exhibited almost the same anti-fatigue performance as that of the TC21 titanium alloy substrate. This is attributed to the polishing-induced residual compressive stress on the surface and a weakening of the notch stress-concentration effect.
(2) Moderate-intensity shot peening can effectively improve the fatigue resistance of the polished HVOF WC-17Co coatings. This is attributed to the deep distribution, high residual compressive stress field, and hardening effect of the coatings.
(3) The HVOF WC-17Co coatings subjected to excessively high intensities of shot peening crack and separate from the substrate, and the residual compressive stress (induced by shot peening) is relaxed. These occurrences are all detrimental to the fatigue resistance.

## Figures and Tables

**Table 1:** Spray parameters of high velocity oxygen fuel (HVOF).

Powder Feed Rate (kg/h)	Carrier Gas Pressure (Mpa)	Oxygen Pressure (Mpa)	Combustion Gas	Combustion Gas Pressure (Mpa)	Spray Distance (mm)	Gun Traverse Speed (mm/s)	Specimen Rotation (r/min)	Temperature of Substrate (°C)
4	1.4	1.5	Propane	1.4	300	200	3000	<150
